# High-Efficiency CsPbBr_3_ Light-Emitting Diodes using One-Step Spin-Coating In Situ Dynamic Thermal Crystallization

**DOI:** 10.3390/mi14061104

**Published:** 2023-05-24

**Authors:** Buyue Zhang, Chen Chen, Xinyu Chen

**Affiliations:** 1 College of Physics, Changchun University of Science and Technology, Changchun 130012, China; 2 College of Information Technology, Jilin Normal University, Siping 136000, China

**Keywords:** all inorganic perovskite, light-emitting devices, low-temperature in situ dynamic thermal crystallization

## Abstract

All-inorganic perovskite materials (such as CsPbBr_3_) have received widespread attention because of their better stability than hybrid counterparts, but their poor film morphology and crystalline quality limit their application in perovskite light-emitting devices (PeLEDs). Some previous studies have attempted to improve the morphology and crystalline quality of perovskite films by heating the substrate, but there are still some problems such as inaccurate temperature control, excessive temperature is not conducive to flexible applications, and the mechanism of action is not clear. In this work, we used a one-step spin-coating, low-temperature in situ thermally assisted crystallization process, in which the temperature was accurately monitored using a thermocouple in the range of 23–80 °C, and explored the effect of the in situ thermally assisted crystallization temperature on the crystallization of the all-inorganic perovskite material CsPbBr_3_ and the performance of PeLEDs. In addition, we focused on the influence mechanism for the in situ thermally assisted crystallization process on the surface morphology and phase composition of the perovskite films and promote its possible application in inkjet printing and scratch coating methods.

## 1. Introduction

Metal halide perovskites have attracted increasing attention due to their exceptional optoelectronic properties such as high charge carrier mobility [1,2,3], narrow emission linewidth [4,5], readily tunable emission wavelengths [6,7], and low cost of solution preparation, which make them ideal for use in optoelectronic applications, including solar cells [8], light-emitting diodes (LEDs) [9,10,11], photodetectors [12], and lasers [13]. Since 2014, PeLEDs have enabled a major breakthrough, especially in the external quantum efficiency (EQE) for organic–inorganic hybrid lead bromide, green-emitting perovskite LEDs, which has recently exceeded 20% [14]. Unfortunately, hybrid perovskites, which contain small organic cations such as methylammonium (MA) or formamidinium (FA), are extremely moisture-sensitive, which leads to rapid degradation in LED performance and thus limits their prospects for practical applications [15,16,17]. Perovskites based on inorganic cesium cations, namely CsPbX_3_ (X = Cl, Br, and I), exhibit better thermal and chemical stability compared to their hybrid analogs, and may thus provide the base for high-performance LEDs with reasonable operational stability [18,19].

Currently, several different methods have been developed for fabricating dense CsPbBr_3_ perovskite films with uniform thickness, such as solution processing methods including one-step spin-coating [20,21], two-step spin-coating [22,23,24,25,26,27], spray coating [28,29,30,31,32], and ink-jet printing [33,34]. In these methods, substrate heating has some preliminary applications in the current research related to perovskite thin films. In 2015, the team of Prof. Aditya D. Mohite at Los Alamos National Laboratory reported the preparation of perovskite films with very large grain sizes using a hot-cast solution deposition process for the first time [35]. In contrast to the conventional spin-coating process for film formation followed by annealing, the hot-cast solution deposition technique coats the heated precursor solution directly onto the substrate at 180 °C. Since the in situ-assisted thermal crystallization temperature is much higher than the perovskite crystallization temperature, and the presence of excess solvent allows the perovskite crystals to grow for a long time, producing a large number of large size crystal particles. However, the process requires a solvent with a high boiling point, thus limiting its application on flexible devices. In 2016, on this basis, Prof. Erik M. J. Johansson’s team at Uppsala University reported a method for preparing perovskite solar cells in air [36]. The researchers spin-coated a heated precursor solution onto a preheated substrate under atmospheric conditions and annealed it to obtain a well-crystallized perovskite film. The results showed that the step of preheating the substrate has an effect on the thickness as well as the crystallinity of the perovskite films, but the substrate preheating temperature above 80 °C leads to poor homogeneity in the perovskite films. The effect of substrate temperature on the crystallization process for perovskite thin films in the one-step method was reported by Prof. Xiaobin Liu’s group at the University of Electronic Science and Technology in 2019 [37]. To accurately control the substrate temperature, the researchers heated the substrate to 150 °C and then quickly spin-coated the solution onto the substrate when it cooled to a predetermined temperature. They found that when the substrate temperature was controlled within a certain range, a highly crystalline perovskite layer could be formed. However, due to the experimental conditions, the temperature of the substrate continued to decrease during the spin-coating process, which affected the accuracy of the experimental analysis. Based on the investigations mentioned above, our team reported that low-temperature in situ dynamic thermal crystallization achieves high-performance CsPbBr_3_-based PeLED. We conducted the annealing process while evaporating the precursor (CsBr and PbBr_2_), and the fabricated devices demonstrated superior performance [38]. Furthermore, the EQE of green PeLED was over 26%, which implied the great potential for the commercializatioin [39].

In this work, we applied the in situ thermally assisted crystallization process to a one-step spin-coating method. The effect of in-situ preparation temperature on the crystallization of CsPbBr_3_ perovskite films during one-step preparation and the performance of PeLEDs were investigated. The substrate was heated continuously while spin-coating the perovskite film, and the temperature was accurately monitored using a thermocouple in the range of 23–80 °C. Using an analysis of the morphology and properties of CsPbBr_3_ films at different temperatures, we focus on the influence mechanism of the in situ thermally assisted crystallization process on the surface morphology and phase composition of the perovskite film and promote its possible application in inkjet printing and scratch coating methods.

## 2. Experimental Section

Materials Preparation. Cesium bromide (CsBr, 99.9%), lead bromide (PbBr_2_, 99.99%), and pre-patterned, indium-doped tin-oxide (ITO) substrates were purchased from Advanced Election Technology Co., Ltd. (Beijing, China), and used without further purification. PEDOT: PSS, 1,3,5-Tris(1-phenyl-1H-benzimidazol-2-yl) benzene synonym (TPBI, 99.5%), 8-hydroxyquinolinolato-lithium (Liq, 99.5%), and aluminum (Al) were purchased from Xi’an Polymer Light Technology Corp. (Xi’an, China) and used without further purification.

Device Fabrication. The ITO-coated substrates (25 mm × 25 mm) were cleaned using sequential sonication in acetone, ethanol, and deionized water for 15 min. in each solvent and then dried in an oven at 100 °C for 30 min. After 20 min UV ozone treatment for ITO, a PEDOT:PSS aqueous solution was spin-coated onto the ITO substrate using a one-step process (4000 rpm for 30 s) and then baked at 150 °C for 20 min in a dry environment. The specific process flow for the one-step spin-coating in situ thermally assisted crystallization process for CsPbBr_3_ is described in Figure S1. The substrates were loaded into a high-vacuum chamber (base pressure ≈ 2 × 10^−6^ torr) to deposit TPBI (50 nm), Liq (1 nm), and Al (100 nm) layer-by-layer. The substrates were kept at room temperature with the sample holder rotating at 30 rpm. All the functional materials were also sequentially deposited using thermal evaporation and growth at a standard deposition rate of 0.6 Å/s without substrate heating.

Characterizations. The CsPbBr_3_ films and PeLEDs were tested in the air without encapsulation. The thickness of each material layer was determined using an oscillating quartz thickness monitor (INFICON SQC-310C) located in the vacuum chamber to determine the deposition rate. Scanning electron microscopy (SEM) was conducted using a JSM-7500F field-emission scanning electron microscope (JEOL) to observe the morphology of the CsPbBr_3_ films. The surface roughness of the CsPbBr_3_ films was measured with atomic force microscopy (AFM) using NX10 atomic force microscopy (Park). X-ray diffraction (XRD) was conducted using a model D/max 2400 X-ray diffraction (Bruker). Photoluminescence (PL) spectra were obtained using an RF-5301PC fluorescence spectrophotometer (SHIMADZU). Electroluminescence (EL) spectra and CIE colorimetry values were collected using a PR655 SpectraScan spectroradiometer (Photo Research). The current-voltage luminance characteristic curves were obtained using a B290222A precision source/measure unit (Agilent, Santa Clara, CA, USA) in the air at room temperature.

## 3. Result and Discussion

The color change in the film is the most visual representation of the film’s surface. Photographs showing the CsPbBr_3_ films prepared at different in situ heat-assisted crystallization temperatures are shown in Figure S2. When the in situ thermally assisted crystallization temperature was increased, the color of the unannealed CsPbBr_3_ film gradually deepened from light to earthy, and the light transmission almost changed from fully transparent to semitransparent. As the in situ dynamic thermal crystallization temperature continued to rise above 70 °C, the CsPbBr_3_ film increased in haze and assumed a frosted state. The reason for the color change here may come from changes in the thickness of the CsPbBr_3_ film and the presence of derivatives of CsPbBr_3_ (e.g., CsPb_2_Br_5_ and Cs_4_PbBr_6_). In addition, the increase in film haze when the in situ heat-assisted crystallization temperature rises above 70 °C may originate from the change in surface roughness.

**Figure 1:**
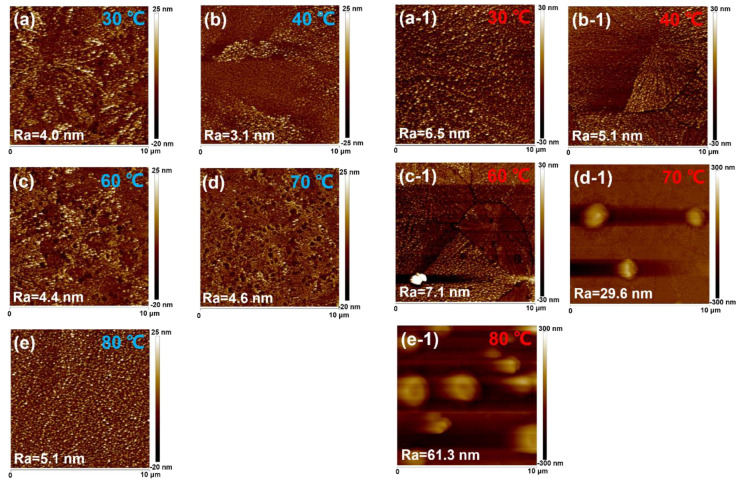
The AFM images showing the CsPbBr_3_ films which were prepared at different in situ thermally assisted crystallization temperatures. (**a**–**e**) Unannealed and (**a1**–**e1**) annealed.

To analyze the effect of in situ thermally assisted crystallization on the film morphology, we characterized the surface roughness of the unannealed CsPbBr_3_ films using atomic force microscopy (AFM). Figure 1a–e shows the height AFM images of unannealed CsPbBr_3_ films prepared at different in situ thermally assisted crystallization temperatures with an image size of 10 × 10 μm. When the in situ thermally assisted crystallization temperature was increased from room temperature to 40 °C, the surface roughness of the films decreased from 4.1 nm to 3.1 nm and showed a dendritic morphology. Therefore, the lower in situ thermally assisted crystallization temperature may not accelerate solvent volatilization to influence the crystallization dynamic. In addition, when the temperature increased to 40 °C, the dendritic morphology on the surface of CsPbBr_3_ films almost disappeared, which indicated that the in situ thermally assisted crystallization temperature of 40 °C improved the agglomeration phenomenon and made the films become flatter and further reduced the surface defects sites of CsPbBr_3_ films. With the increase in temperature to 60 °C and 70 °C (as shown in Figure 1c,d), it can be seen more obviously that the pin-holes on the surface of the films increased rapidly and that island-like morphology started to be generated and increased significantly with the increase in temperature, while the surface roughness of the unannealed CsPbBr_3_ films increased from 3.1 nm to 4.6 nm. This implies that although the in situ thermally assisted crystallization process can accelerate the solvent volatilization to reduce perovskite agglomeration, once the evaporation rate is too fast, it will in turn lead to the increase in pin-holes on the films. These pin-holes existing among the PeLED devices may cause direct contact between the cathode and anode. Meanwhile, like grain boundaries, the pin-holes may contain many handing bonds acting as defect sites, which could cause serious nonradiative recombination. When the in situ thermally assisted crystallization temperature was further increased to 80 °C, the island-like morphology on the surface of the unannealed CsPbBr_3_ film became more obvious, accompanied by an enhancement in Ra, which may be due to the fact that the in situ thermally assisted crystallization temperature of 80 °C greatly accelerated the volatilization rate of the DMSO solvent, at which time the CsPbBr_3_ rapidly pre-crystallized around the nucleation site under the unannealed crystallization condition.

Figure 1a1–e1 shows that the AFM of CsPbBr_3_ films after annealing at different in situ thermally assisted crystallization temperatures provide the morphology of the annealed CsPbBr_3_ films. When the in situ thermally assisted crystallization temperature is below 60 °C, the surface of the films is relatively flat with Ra not exceeding 10 nm, while when the in situ thermally assisted crystallization temperature exceeds 70 °C, the surface of the CsPbBr_3_ films shows bumps of up to several hundred nanometers with a diameter of about 1 μm. It is also noted that when the in situ thermally assisted crystallization temperature rises to 80 °C, the bumps are denser, and the surface roughness of the film is larger. The generation of island-like morphology in perovskite electroluminescent devices can directly lead to contact between the perovskite layer and the electrode, resulting in carrier tunneling and destruction of the device. In addition, the laser confocal micrographs showing the unannealed and annealed CsPbBr_3_ films (Figure S4) at different in situ thermally assisted crystallization temperatures also show similar trends, which further support our conclusion.

In Figure S3, we summarize the surface roughness variation trend in CsPbBr_3_ films after annealing. It is found that the change in surface roughness of the films is small when the in situ thermally assisted crystallization temperature is below 60 °C. The in situ thermally assisted crystallization temperature above 60 °C accelerates the volatilization rate of the DMSO solvent, which leads to the growth of the films around the nucleation as sites. Therefore, for CsPbBr_3_ films prepared using DMSO solvent, an in situ thermally assisted crystallization temperature of 60 °C is the critical temperature for obtaining smooth CsPbBr_3_ films. Combining Figure 1b1,c1 to compare the surface morphology of the films prepared at the in situ thermally assisted crystallization temperatures of 40 °C and 60 °C, we can see that the in situ thermally assisted crystallization temperature of 40 °C results in lower surface grain boundary density, a lower density of defect states, and fewer pin-holes in the CsPbBr_3_ films, which may theoretically be the key to obtaining higher performance LEDs. The annealing temperature plays an important role in the variation in the morphology of the perovskite film. The elevated temperature may accelerate the evaporation of the solvent to influence the dynamics of crystallization. So, we attributed the disappearance of pin-holes to the difference in the annealing temperature.

According to the previous section, the surface morphology of CsPbBr_3_ films using the in situ thermally assisted crystallization process was improved, and the defect sites were significantly reduced. To further understand the reasons for this, the structure of the CsPbBr_3_ films prepared using the in situ thermally assisted crystallization process was analyzed.

**Figure 2:**
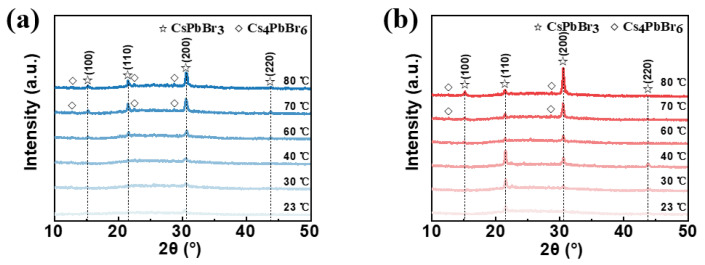
XRD patterns for the (**a**) unannealed and (**b**) annealed CsPbBr_3_ films prepared at different in situ thermally assisted crystallization temperatures.

Firstly, the unannealed and annealed CsPbBr_3_ films prepared at different in situ thermally assisted crystallization temperatures were characterized using X-ray diffraction (XRD) as shown in Figure 2a,b, respectively. As shown in Figure 2a, the intensity of the diffraction peak at the 2θ angle of 30.5°, which corresponds to the (200) crystal face of CsPbBr_3_, gradually increases when increasing the in situ thermally assisted crystallization temperature of the unannealed CsPbBr_3_ film. When the in situ thermally assisted crystallization temperature is above 60 °C, two diffraction peaks at 15.2° and 21.4° appear, corresponding to the (100) and (110) crystallographic planes of CsPbBr_3_, respectively. At the same time, the intensity of these two diffraction peaks increased with the increase in temperature. When the temperature rises to 70 °C and above, the films show diffraction peaks of Cs_4_PbBr_6_ at 12.7°, 22.4° and 28.6°. This is probably due to the fact that at a certain temperature, the unreacted CsBr in the precursor solution reacts with the CsPbBr_3_ first produced during the spin-coating process to form Cs_4_PbBr_6_. The reaction principle is shown in Equation (1). The increase in surface roughness of the films may therefore come from the production of Cs_4_PbBr_6_.

CsPbBr_3_ + 3CsBr = Cs_4_PbBr_6_ (1)

Since Cs_4_PbBr_6_ is easily converted to CsPbBr_3_ with thermal instability, we characterized the annealed CsPbBr_3_ films using XRD. The CsPbBr_3_ diffraction peaks are at 12.7° and 30.5°, which gradually increase in intensity as the temperature increases. The diffraction peak in Cs_4_PbBr_6_ disappears at 22.4°, which is mainly due to the conversion of Cs_4_PbBr_6_ into CsPbBr_3_ during annealing. It is noteworthy that the diffraction peak at 21.4° corresponding to the CsPbBr_3_(110) crystal plane increases in intensity as the temperature rises from 23 °C to 40 °C, while as the temperature rises from 40 °C to 80 °C, the intensity of the diffraction peak at the same position decreases instead. The CsPbBr_3_(220) crystal plane diffraction peak at 43.8° has almost the same behavior. Related studies suggest that CsPbBr_3_ films grown along the (110) direction have better EL properties [38]. Therefore, we found that the in situ thermally assisted crystallization process has a strong influence on the crystallographic orientation of the crystals in the one-step preparation of perovskite films and that high in situ thermally assisted crystallization temperatures for derivative-prone perovskite materials can lead to the formation of derivatives.

**Figure 3:**
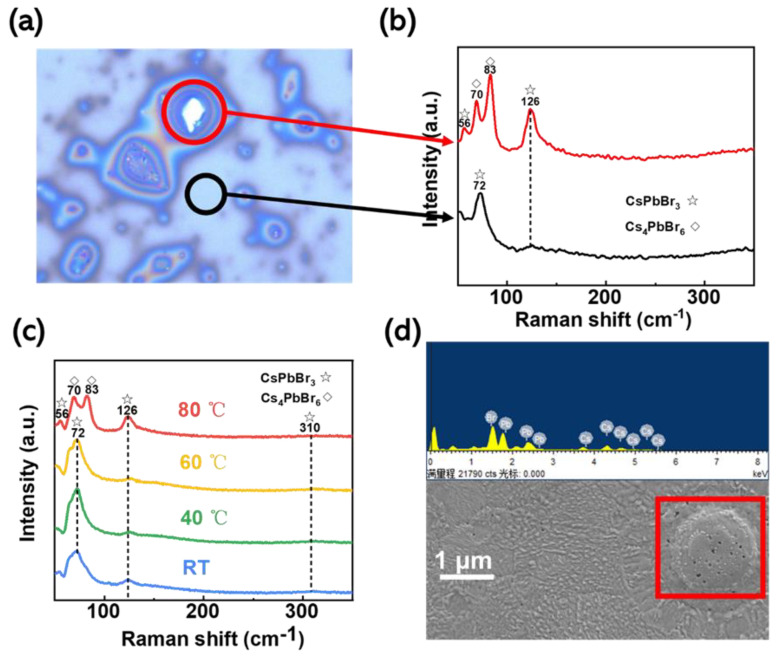
(**a**) Raman micrograph showing the surface of the CsPbBr_3_ film after annealing at an in situ thermally assisted crystallization temperature of 80 °C. The red circle shows the island-like morphology described above, and the black circle shows the non-island-like morphology. Respectively with (**b**), when the in situ thermally assisted crystallization temperature is 80 °C, the red line and the black line in the Raman spectrum for the film sample correspond; (**c**) are the Raman spectra for the CsPbBr_3_ film samples at different in situ heat-assisted crystallization temperatures; (**d**) are the SEM images showing the surface of the CsPbBr_3_ film. The red box is the scanning area of EDS mapping, where the EDS map is above (**d**).

To verify that the island-like morphology is due to the Cs_4_PbBr_6_ crystals, we tested the Raman spectra of the corresponding positions. Figure 3a shows a Raman micrograph for in situ thermally assisted crystallization at a temperature of 80 °C. The red circles show the island-like morphology described previously, and the black circles show the non-island-like morphology. The island-like (red circle) and non-island-like (black circle) features in Figure 3b correspond to the characteristic Raman peaks in CsPbBr_3_ and Cs_4_PbBr_6_, respectively. There are three more significant Raman peaks for CsPbBr_3_ in the figure, a weaker shoulder peak at 56 cm^−1^ and two weak broad peaks at 126 cm^−1^ and 310 cm^−1^, where 56 cm^−1^ and 126 cm^−1^ correspond to the vibrational mode of the [PbBr_6_]^4−^ normal octahedron and the motion mode of the Cs^+^ ion, and 310 cm^−1^ corresponds to the second-order phonon model for the normal octahedron. In contrast, Cs_4_PbBr_6_ has strong characteristic Raman peaks at 70 cm^−1^, 83 cm^−1^, and 126 cm^−1^, corresponding to the [PbBr_6_]^4−^ ortho-octahedral mode of vibration. Notably, the disappearance of the theoretical strongest characteristic peak position in CsPbBr_3_ at 72 cm^−1^, as shown in the Figure, thus proves that the bulge on the film surface is produced by both CsPbBr_3_ and Cs_4_PbBr_6_. Considering the temperature sensitivity of Cs_4_PbBr_6_, the characteristic Raman peak in CsPbBr_3_ at 56 cm^−1^, shown in the red sample bar in Figure 3b, may come from the partial conversion of Cs_4_PbBr_6_ into CsPbBr_3_.

The films prepared at different in situ thermally assisted crystallization temperatures were also characterized using Raman spectroscopy, as shown in Figure 3c. Only when the temperature reached 80 °C did the characteristic Raman peak in Cs_4_PbBr_6_ appear. This also demonstrates that the in situ thermally assisted crystallization temperature accelerates the volatilization rate of DMSO, but the excessively fast crystallization rate leads to preferential growth of CsPbBr_3_ at some locations on the substrate surface and results in the production of CsPbBr_3_ derivatives on this basis. The results of our energy spectroscopy (EDS) analysis of the island-like morphology on the film surface are shown in Figure 3d. We have carried out an EDS surface scan analysis of the island-like formations at the red squares, and it can be seen from Table S1 that the atomic percentages of Cs, Pb, and Br are 35.36%, 8.22%, and 56.42%, respectively, with elemental ratios close to 4:1:6, which is a ratio consistent with the elemental ratio in the Cs_4_PbBr_6_ molecular formula. The island-like morphology is more likely to be Cs_4_PbBr_6_.

**Figure 4:**
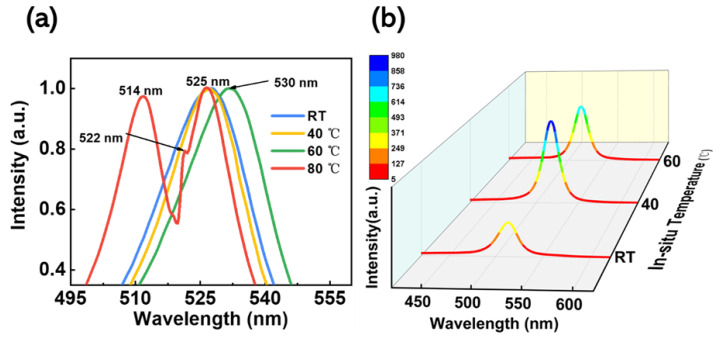
(**a**) Steady-state normalized photoluminescence spectra showing the CsPbBr_3_ films at different in situ thermally assisted crystallization temperatures. (**b**) Steady-state photoluminescence spectra showing the CsPbBr_3_ film at 23 °C, 40 °C, and 60 °C in situ thermally assisted crystallization.

To further demonstrate that the in situ thermally assisted crystallization process can suppress defect states in perovskite films, steady-state fluorescence (PL) spectra, as well as transient fluorescence (TRPL) spectra, were tested, both at room temperature with an excitation light wavelength of 365 nm. Figure 4a shows the steady-state normalized photoluminescence spectra of the films at different in situ thermally assisted crystallization temperatures. As can be seen in the figure, when the in situ thermally assisted crystallization temperature was below 40 °C, the films exhibited a PL peak in CsPbBr_3_ at 525 nm, and the full width at half maxima (FWHM) was narrower at 40 °C compared to the films prepared at 23 °C, indicating better crystallinity of the CsPbBr_3_ films at 40 °C. When the temperature rises to 60 °C, the PL peak in the film shows a significant red-shift, which, for perovskite films, is related to surface defect states and grain boundaries. Moreover, when the in situ thermally assisted crystallization temperature was higher than 60 °C, the crystallization of the prepared perovskite films was worse, which is consistent with the results discussed earlier. When the temperature increased to 80 °C, a PL luminescence peak at 514 nm Cs_4_PbBr_6_ can be found in the film, together with a shoulder peak at 522 nm. The appearance of the blue-shifted shoulder peak here is related to the quantum-limited effect. The reaction of CsPbBr_3_ with CsBr leads to a reduction in the crystal size of CsPbBr_3_ nested in the crystals of Cs_4_PbBr_6_ [40]. This supports the view that the characteristic CsPbBr_3_ Raman peaks in the Raman spectral analysis in the previous section may be due to the partial conversion of Cs_4_PbBr_6_ into CsPbBr_3_. Figure 4b shows the steady-state photoluminescence spectra of the films at in situ thermally assisted crystallization temperatures of 23 °C, 40 °C, and 60 °C. It can be seen that the PL intensity at 40 °C is the strongest, even higher than that at 60 °C where the film thickness is thicker, which proves that the films prepared using the in situ thermally assisted crystallization process at 40 °C have fewer defect sites and higher crystalline quality [41].

**Figure 5:**
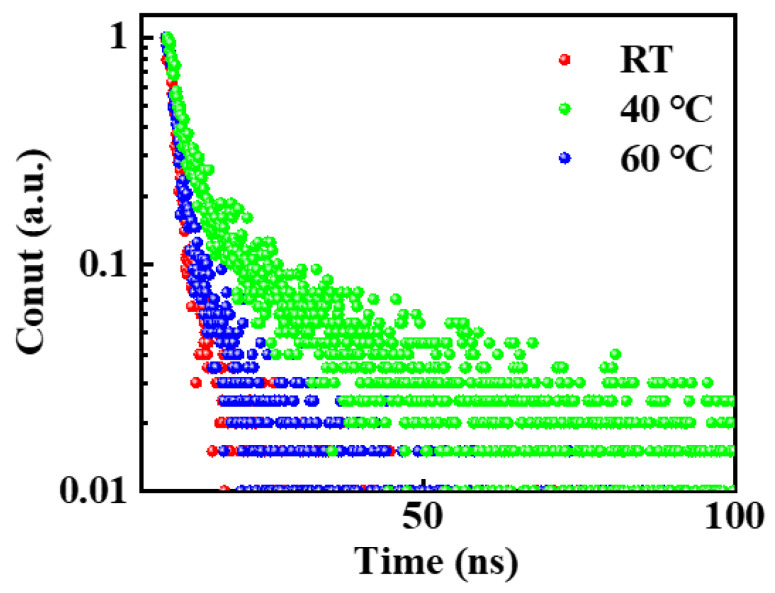
Time-resolved photoluminescence spectra showing the CsPbBr_3_ films at different in situ thermally assisted crystallization temperatures.

In addition to the degree of crystallinity and crystal orientation in the CsPbBr_3_ films grown at different temperatures, the nature of defects in the film is also a critical feature that affects the properties of the photo-generated carriers and PeLEDs performance with the device structure of ITO/PEDOT:PSS/perovskite. Therefore, the trap states of the CsPbBr_3_ films were investigated by evaluating their time-resolved photoluminescence (TRPL), which is presented in Figure 5. In addition, the TRPL curves in Figure 5 were fitted using biexponential decay functions, and the average decay times for the two components in each of the biexponential decay functions included a relatively short lifetime *τ*_1_ with an area under the TRPL curve of *A*_1_ and a longer lifetime *τ*_2_ with an area under the TRPL curve of *A*_2_. The average decay time *τ_ave_* was then calculated using the following expression:

(2) $$\tau_{ave}=\frac{A_{1}\tau_{1}{}^{2}+A_{2}\tau_{2}{}^{2}}{A_{1}\tau_{1}+A_{2}\tau_{2}}$$

The charge carrier lifetimes obtained for the CsPbBr_3_ films grown at RT, 40 °C, and 60 °C are listed in Table 1. The TRPL spectra show two characteristic decays. The faster decay, associated with lifetime *τ*_1_, is related to trap-assisted non-radiative recombination by defects sites in the perovskite film, especially on the surface of perovskite grains with a higher defect concentration. The slower decay, associated with lifetime *τ*_2_, is related to radiation recombination of the excited states inside the perovskite grains. Compared with room temperature, the carrier lifetime under the in situ thermally assisted crystallization temperature of 40 °C is slightly improved, where *τ*_1_ increased from 1.57 ns to 1.59 ns and *τ*_2_ increased from 13.05 ns to 14.03 ns. The increase in *τ*_1_ indicates that defect states on the surface of the CsPbBr_3_ film are reduced, resulting in less non-radiation recombination. On the other hand, the increase in *τ*_2_ demonstrates a longer carrier lifetime and indicates the improved quality of the CsPbBr_3_ film. However, when the in situ thermally assisted crystallization temperature is 60 °C, the carrier lifetime will be significantly decreased, where *τ*_1_ decreased from 1.59 ns to 1.30 ns and *τ*_2_ decreased from 14.03 ns to 8.38 ns. This shows that when the in situ heat-assisted crystallization temperature is too high, the defect density increases, leading to the internal radiation recombination decrease, and the quality of the CsPbBr_3_ film relatively decreases. Overall, the average lifetime of the CsPbBr_3_ film prepared at the 40 °C in situ thermally assisted crystallization temperature is 5.87 ns, which is significantly longer than the 4.41 ns lifetime of the sample prepared at 23 °C. This result can be explained by the observed reduction in grain boundary trap-assisted recombination. Meanwhile, the results for PLQY in Figure S5 were inconsistent with the TRPL measurement.

In order to gain a deeper understanding of the reasons for the enhancement in the PLQY of films prepared using the in situ thermally assisted crystallization process, here we use the space-charge-limited current (SCLC) method to quantify and compare the changes in defects density with and without the in situ thermally assisted crystallization processes. We prepared a single-hole carrier device with the structure ITO/PEDOT:PSS/CsPbBr_3_/CBP/MoO_3_/Ag.

**Figure 6:**
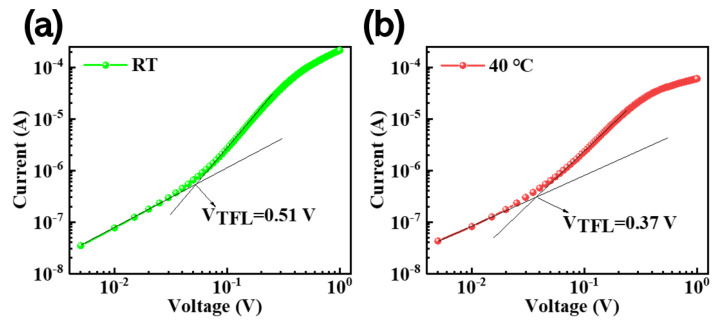
(**a**,**b**) The SCLC diagrams for the single-hole carrier device prepared using CsPbBr_3_ thin film at RT and the 40 °C in situ thermally assisted crystallization temperature, respectively.

From the I-V curve in Figure 6, three different areas can be clearly identified, including Ohmic, Trap Filled Limit, and SCLC. We calculated the hole and electron defect state density (*N_t_*) for the CsPbBr_3_ film using a single-hole carrier device. The calculation method is carried out according to the following formula:

(3) $$N_{t}=\frac{2{\varepsilon \varepsilon }_{0}V_{TFL}}{{\mathrm{eL}}^{2}}\;$$

where ε and ε_0_ represent the relative permittivity and vacuum permittivity of the perovskite, respectively (8.854 × 10^−12^ F/m), *V_TFL_* is the defect limit voltage (the defect state is completely restricted within this voltage range), and L represents film thickness, where the thickness of the CsPbBr_3_ film is obtained using a step meter test and e represents the elementary charge. The thickness of the CsPbBr_3_ film at different in situ heat-assisted crystallization temperatures is shown in Table S1. Therefore, we can calculate the defect state density for the perovskite film prepared at room temperature and the 40 °C in situ thermally assisted crystallization temperature as: 3.59 × 10^16^ cm^−3^ and 2.3 × 10^16^ cm^−3^, respectively. The defect state density for the film at the in situ heat-assisted crystallization temperature of 40 °C is significantly reduced.

**Figure 7:**
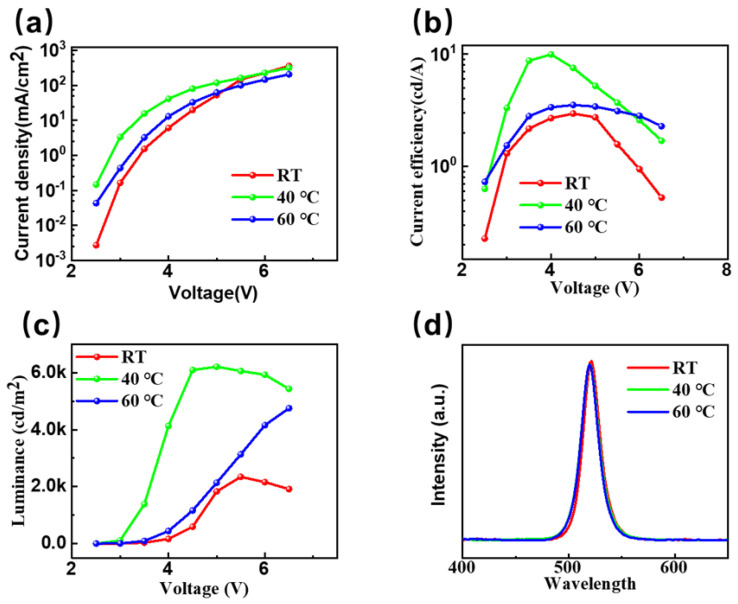
EL properties of CsPbBr_3_ prepared at RT and the 40 °C and 60 °C in situ thermally assisted crystallization temperatures. (**a**) Current density–voltage characteristic curve; (**b**) current efficiency–voltage characteristic curve; (**c**) brightness–voltage characteristic curve; and (**d**) normalized electroluminescence spectra for CsPbBr_3_ light emitting devices prepared at different in situ thermally assisted crystallization temperature work on 5 V driving voltage.

By characterizing the electroluminescence properties of CsPbBr_3_ PeLEDs prepared under different in situ thermally assisted crystallization temperatures using one-step spin-coating, we can further analyze the in situ thermally assisted crystallization temperature and its influence on the properties of CsPbBr_3_ films. Figure 7a shows the current density–voltage characteristic curve for the electroluminescent device at RT and the 40 °C and 60 °C in situ thermally assisted crystallization temperatures. We found that when the in situ thermally assisted crystallization temperature is 40 °C, with the same operating voltage, the device exhibits the highest current density. Combined with our previous research results on the effect of in situ thermally assisted crystallization temperature on the morphology of CsPbBr_3_ films, it is further proved that CsPbBr_3_ films prepared using the low-temperature in situ thermally assisted crystallization process have fewer defects. When the same number of carriers are injected, the proportion of non-radiative recombination generated when filling defects is less. When the in situ heat-assisted crystallization temperature is 40 °C, its thickness is not much different from that of the CsPbBr_3_ film prepared at RT. Therefore, compared with the film prepared at 60 °C, the radiation recombination rate of the device is improved with the limitation in the carrier space, so it shows a higher current density. Figure 7b shows the current efficiency–voltage characteristic curve of PeLEDs at RT and the 40 °C and 60 °C in-situ thermally assisted crystallization temperatures. From the figure, we can see that at 40 °C, the current efficiency of the device is as high as 9.9 cd/A, which is almost 3 times (RT, 3.1 cd/A) that seen when the process method is not used. In Figure 7c, we can see that when the driving voltage is only 5 V at 40 °C, the device has reached a brightness of 6208 cd/m^2^, while it is only 1829 cd/m^2^ at RT. This further proves that the low-temperature in-situ thermal-assisted crystallization technology improves the surface morphology of the CsPbBr_3_ film, reduces surface defects, and therefore reduces the non-radiative recombination rate. Figure 7d shows the electroluminescence spectra for CsPbBr_3_ electroluminescent devices prepared at RT and the 40 °C and 60 °C in situ thermally assisted crystallization temperatures. All spectra show the equivalent luminescence peak position at 520 nm, which corresponds to the CIE color coordinate (0.11, 0.77). It shows that the low-temperature in-situ heat-assisted crystallization process has almost no effect on the position of the emission peak in the CsPbBr_3_ electroluminescent device. Meanwhile, the EQE measurement shown in Figure S6 also proved that the 40 °C in situ thermally assisted crystallization temperature delivered the best-performance device.

## 4. Conclusions

In this manuscript, we systematically investigated the effect of a one-step spin-coating method combined with the in situ thermally assisted crystallization process on the defect density for states of CsPbBr_3_. The results confirm that the surface defect density for states of CsPbBr_3_ films can be reduced using a low temperature, in situ thermally assisted crystallization process, which reduces the non-radiative composite rate. On this basis, we prepared CsPbBr_3_ light-emitting devices that can reach a maximum brightness of 6208 cd/m^2^ at 5 V. A maximum current efficiency of 9.9 cd/A can be obtained at the same time. Furthermore, for the light-emitting devices without the low temperature in situ thermally assisted crystallization process, an almost 3-fold increase in brightness and current efficiency was demonstrated. This result is one of the highest efficiencies ever achieved for an all-inorganic CsPbBr_3_ light-emitting device without additives.

Compared with the previous high-temperature, in situ thermal preparation process often used in the spin-coating process, we demonstrate that the introduced low-temperature, in situ thermally assisted crystallization process can effectively enhance the optoelectronic properties of PeLEDs without other adjacent functional layers. With flexible screen devices being mainstream for future display and lighting devices, the low-temperature, in-situ thermally assisted crystallization process proposed in this work further reduces the requirement for substrate temperature resistance performance and provides a new idea for the solution-based preparation of perovskite films.

## Figures and Tables

**Table 1:** Carrier lifetimes for the CsPbBr_3_ films at different in situ thermally assisted crystallization temperatures.

	RT	40 °C	60 °C
A_1_	203.19	202.19	161.47
*τ*_1_	1.57	1.59	1.30
f_1_	75.2%	63.7%	48.8%
A_2_	8.05	15.20	26.28
*τ*_2_	13.05	14.03	8.38
f_2_	24.8%	36.3%	51.2%
Average *τ*	4.41	5.87	4.92

## Data Availability

Not applicable.

## Supplementary Materials

The following supporting information can be downloaded at: https://www.mdpi.com/article/10.3390/mi14061104/s1, Figure S1. The fabrication process of one-step spin-coating in-situ thermally assisted crystallization process. Figure S2. The color variation of perovskite film at different in situ heat-assisted crystallization temperatures. Figure S3. The roughness variation of perovskite film under different annealing temperature. Figure S4. The laser confocal micrographs of unannealed and annealed CsPbBr_3_ films at different in situ thermally assisted crystallization temperatures. Figure S5. PLQY measurement of the perovskite film under different annealing tem-perature. Figure S6. EQE measurement for the best performance PeLED device. Table S1. The concentration of elements in the perovskite film (red circle).
