# Supplementary material for: High-Efficiency CsPbBr3 Light-Emitting Diodes using One-Step Spin-Coating In Situ Dynamic Thermal Crystallization
Source: Micromachines (Basel). 2023 May 24;14(6):1104. doi: 10.3390/mi14061104 (PMC10300846; doi:10.3390/mi14061104)
Supplement: Supplementary file 1 [file micromachines-14-01104-s001.zip › micromachines-2379580-supplementary.pdf]

micromachines

Supplementary Materials

High-Efficiency $CsPbBr_3$ Light-Emitting Diodes using One-Step Spin-Coating In Situ Dynamic Thermal Crystallization

Buyue Zhang [1], Chen Chen [2] and Xinyu Chen [1,*]

[1] College of Physics, Changchun University of Science and Technology, Changchun 130012, China; 2020200002@mails.cust.edu.cn
[2] College of Information Technology, Jilin Normal University, Siping 136000, China; chenchen@jlnu.edu.cn
* Correspondence: chenxinyucust@163.com

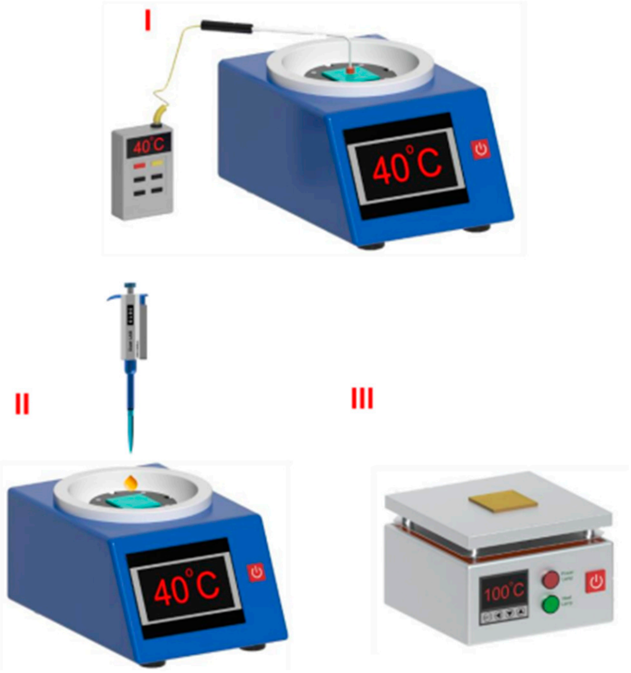

Figure S1. The fabrication process of one-step spin-coating in-situ thermally assisted crystallization process.

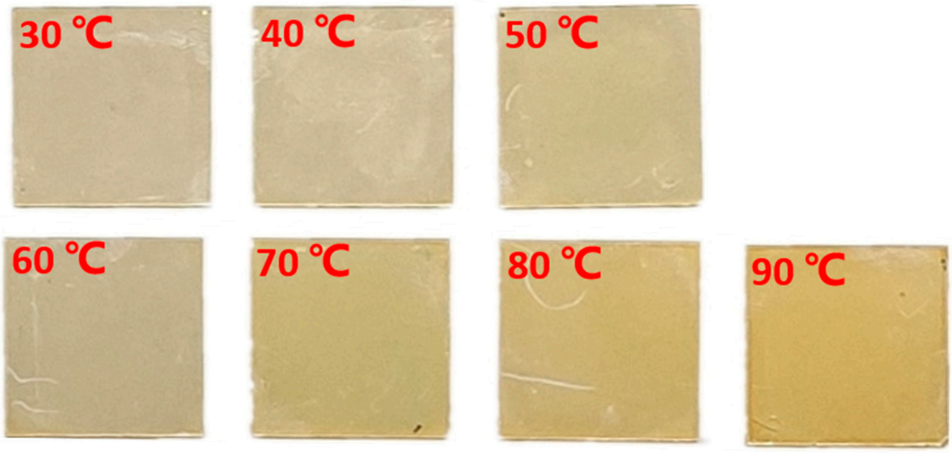

Figure S2. The color variation of perovskite film at different in situ heat-assisted crystallization temperatures.

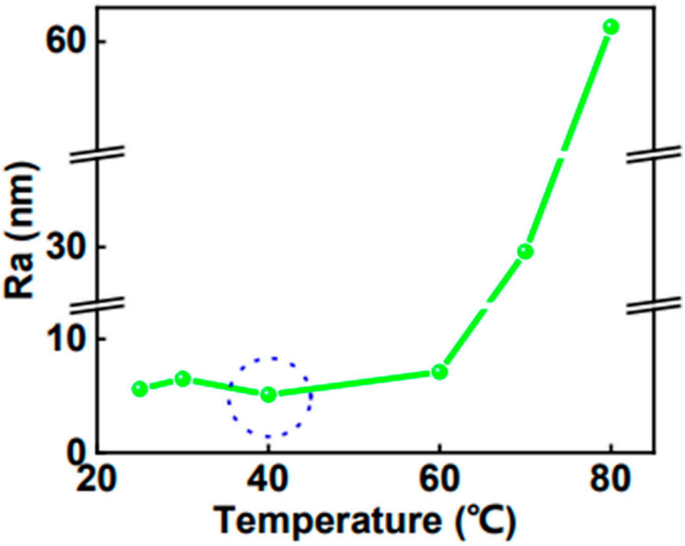

Figure S3. The roughness variation of perovskite film under different annealing temperature.

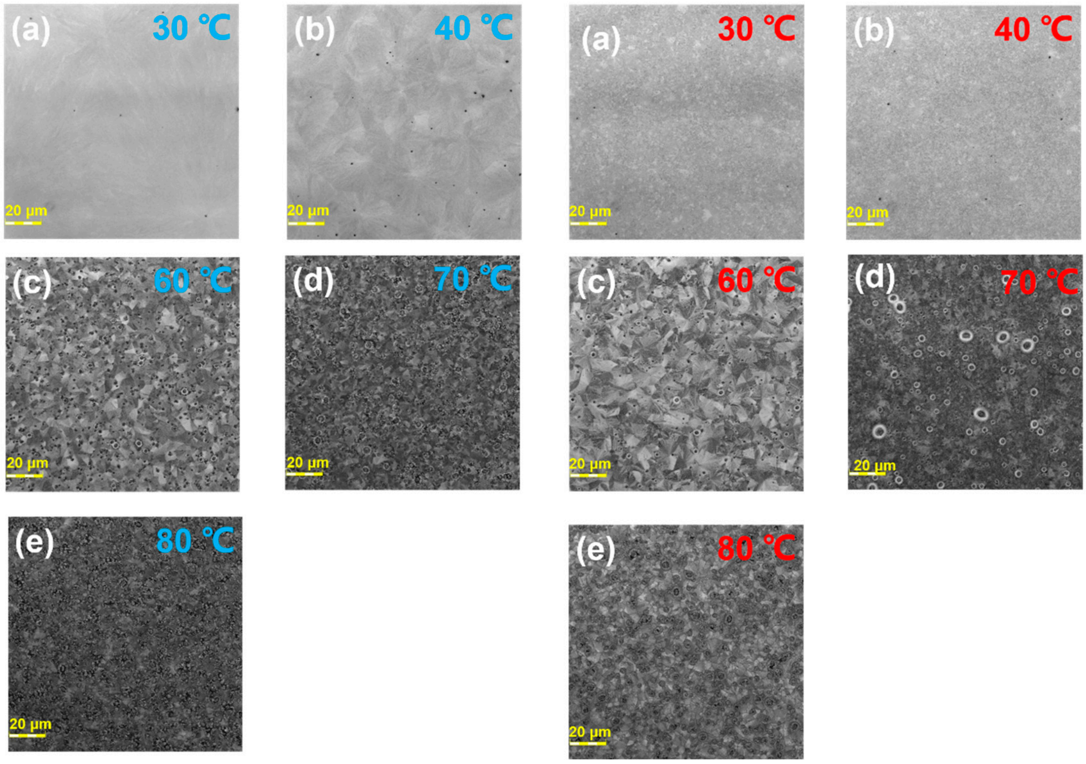

Figure S4. The laser confocal micrographs of unannealed and annealed $CsPbBr_3$ films at different in situ thermally assisted crystallization temperatures.

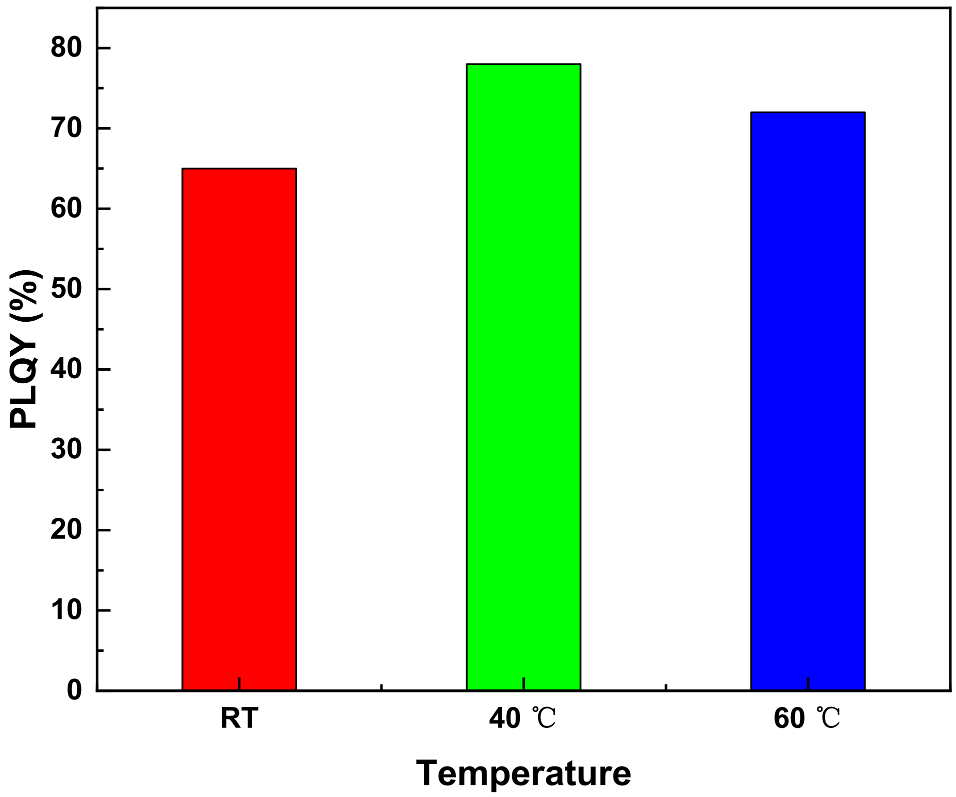

Figure S5. PLQY measurement of the perovskite film under different annealing temperature.

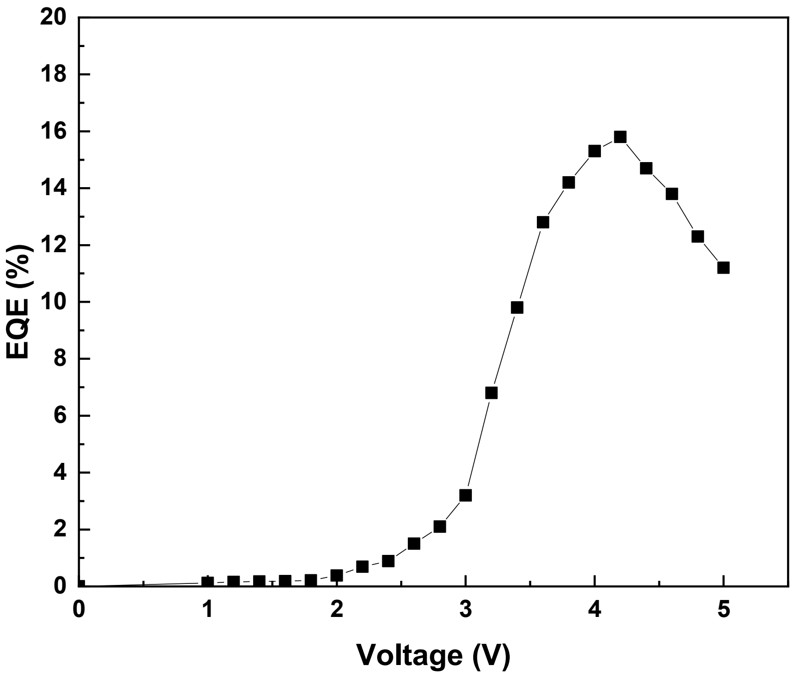

Figure S6. EQE measurement for the best performance PeLED device.

Table S1. The concentration of elements in the perovskite film.

**Element**	**Percentage**
Cs	35.36%
Pb	8.22 %
Br	56.42 %
